# Enoxaparin sodium bone cement plays an anti-inflammatory immunomodulatory role by inducing the polarization of M2 macrophages

**DOI:** 10.1186/s13018-023-03865-8

**Published:** 2023-05-23

**Authors:** Weiye Fan, Dehao Fu, Li Zhang, Zhihang Xiao, Xiaoyu Shen, Jianchao Chen, Xiangbei Qi

**Affiliations:** 1 Department of Orthopaedic Surgery, The Third Hospital of Hebei Medical University, Shijiazhuang, 050035 People’s Republic of China; 2 Department of Orthopedics, Shanghai General Hospital, Shanghai Jiao Tong University School of Medicine, Shanghai, People’s Republic of China

**Keywords:** Bone cement, Inflammation, Enoxaparin sodium, Macrophage

## Abstract

**Objective:**

The implantation of PMMA bone cement results in an immune response and the release of PMMA bone cement particles causes an inflammatory cascade. Our study discovered that ES-PMMA bone cement can induce M2 polarization of macrophages, which has an anti-inflammatory immunomodulatory effect. We also delved into the molecular mechanisms that underlie this process.

**Methods:**

In this study, we designed and prepared samples of bone cement. These included PMMA bone cement samples and ES-PMMA bone cement samples, which were implanted into the back muscles of rats. At 3, 7, and 14 days after the operation, we removed the bone cement and a small amount of surrounding tissue. We then performed immunohistochemistry and immunofluorescence to observe the polarization of macrophages and the expression of related inflammatory factors in the surrounding tissues. The RAW264.7 cells were exposed to lipopolysaccharide (LPS) for 24 h to establish the macrophage inflammation model. Then, each group was treated with enoxaparin sodium medium, PMMA bone cement extract medium, and ES-PMMA bone cement extract medium, respectively, and cultured for another 24 h. We collected cells from each group and used flow cytometry to detect the expressions of CD86 and CD206 in macrophages. Additionally, we performed RT-qPCR to determine the mRNA levels of three markers of M1 macrophages (TNF-α, IL-6, iNOS) and two M2 macrophage markers (Arg-1, IL-10). Furthermore, we analyzed the expression of TLR4, p-NF-κB p65, and NF-κB p65 through Western blotting.

**Results:**

The immunofluorescence results indicate that the ES-PMMA group exhibited an upregulation of CD206, an M2 marker, and a downregulation of CD86, an M1 marker, in comparison to the PMMA group. Additionally, the immunohistochemistry results revealed that the levels of IL-6 and TNF-α expression were lower in the ES-PMMA group than in the PMMA group, while the expression level of IL-10 was higher in the ES-PMMA group. Flow cytometry and RT-qPCR analyses revealed that the expression of M1-type macrophage marker CD86 was significantly elevated in the LPS group compared to the NC group. Additionally, M1-type macrophage-related cytokines TNF-α, IL-6, and iNOS were also found to be increased. However, in the LPS + ES group, the expression levels of CD86, TNF-α, IL-6, and iNOS were decreased, while the expression of M2-type macrophage markers CD206 and M2-type macrophage-related cytokines (IL-10, Arg-1) were increased compared to the LPS group. In comparison to the LPS + PMMA group, the LPS + ES-PMMA group demonstrated a down-regulation of CD86, TNF-α, IL-6, and iNOS expression levels, while increasing the expression levels of CD206, IL-10, and Arg-1. Western blotting results revealed a significant decrease in TLR4/GAPDH and p-NF-κB p65/NF-κB p65 in the LPS + ES group when compared to the LPS group. Additionally, the LPS + ES-PMMA group exhibited a decrease in TLR4/GAPDH and p-NF-κB p65/NF-κB p65 levels when compared to the LPS + PMMA group.

**Conclusion:**

ES-PMMA bone cement is more effective than PMMA bone cement in down-regulating the expression of the TLR4/NF-κB signaling pathway. Additionally, it induces macrophages to polarize towards the M2 phenotype, making it a crucial player in anti-inflammatory immune regulation.

**Graphical Abstract:**

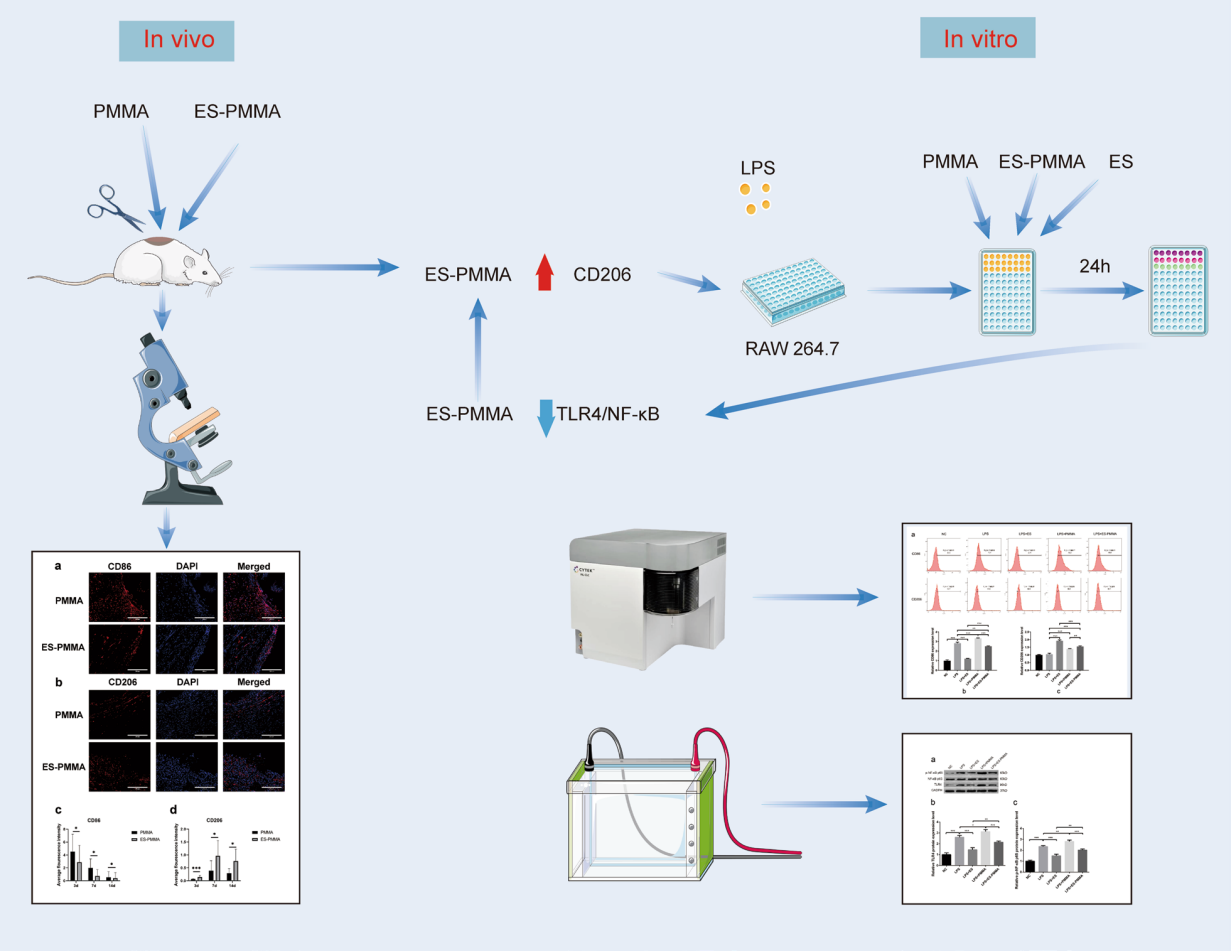

**Supplementary Information:**

The online version contains supplementary material available at 10.1186/s13018-023-03865-8.

## Background

Polymethyl methacrylate (PMMA) bone cement is a commonly used biological material to fill and implant space in vertebroplasty, knee and hip replacement, and repair of large bone defects. This material offers the benefits of strong plasticity and rapid curing [1, 2]. However, it is important to note that the implanted bone cement will inevitably trigger an immune response and the PMMA bone cement particles can lead to an inflammatory cascade [3–5]. The process of tissue healing is characterized by three distinct physiological responses: inflammation, proliferation, and remodeling [6]. Inflammation, in particular, plays a crucial role in the healing process. However, excessive inflammation or a lack of inflammation during the healing process, as well as long-term chronic inflammation, can have detrimental effects on the healing process [7, 8]. It is important to note that macrophages, which are a vital component of the human immune system [9], are an essential part of tissue healing. Macrophages are essential in the process of inflammation, as they are responsible for initiating, maintaining, and resolving it. They carry out three primary functions: presenting antigens, phagocytosis, and regulating the immune system by producing a range of cytokines and growth factors [10]. Macrophages exhibit considerable heterogeneity and plasticity, with their phenotype and function being influenced by environmental signals. They can be polarized into two distinct types: M1 macrophages and M2 macrophages. M1 macrophages are primarily responsible for initiating and sustaining the inflammatory response, while M2 macrophages are primarily involved in resolving inflammation [11]. Research has indicated that the seamless shift of macrophages from M1 to M2 is a vital factor in the onset and resolution of inflammation [12].

In a study conducted by Sun et al. [13], it was found that by mixing 40 g of PMMA bone cement with 8000AxaIU of enoxaparin sodium (ES), a new enoxaparin sodium bone cement (ES-PMMA) could be created. This innovative bone cement has a significantly improved sustained release ability, meaning that the drug release amount can reach the therapeutic amount for thrombosis within 24 h and the preventive amount within 48 h. In their study, Sang et al. [14] showed that ES-PMMA bone cement can effectively reduce local thrombosis by suppressing the expression of CD40 protein in vascular endothelial cells. Although current research on ES-PMMA bone cement has primarily focused on its local anticoagulation properties, it is worth noting that several studies have also demonstrated the anti-inflammatory effects of low molecular weight heparin. In their study, Litov et al. [15] revealed an anti-inflammatory mechanism of low molecular weight heparin (LMWH) by showcasing its impact on the activity of two crucial cytokines, IFNγ and IL-6. Similarly, Wu et al. [16] discovered that LMWH could alleviate the inflammatory state of rats with acute sinusitis by inhibiting the TLR4-MyD88-NF-κB signaling pathway. A study conducted by Abbadi et al. [17] demonstrated that heparin obstructed the polarization of pro-inflammatory macrophages and encouraged the polarization of anti-inflammatory macrophages during hyperglycemic stress. As a result, we hypothesize that ES-PMMA bone cement has the potential to stimulate the polarization of M2 macrophages, alter the discharge of associated inflammatory cytokines, and ultimately perform an immune regulatory function in the nearby tissue microenvironment by reducing inflammation. To test our hypothesis, we implanted bone cement samples into the back muscles of rats and then stained the tissues around the cement using immunohistochemistry and immunofluorescence techniques. In addition, we created an inflammation model by treating RAW264.7 cells with LPS. We then cultured RAW264.7 macrophages using ES and two different extracts from the bone cement samples after treatment. The expression levels of macrophage markers and secreted inflammatory factors in each group were detected by flow cytometry, RT-qPCR, and Western blot. The specific function of ES-PMMA bone cement in macrophage polarization was explored, and the anti-inflammatory immune regulation effect of ES-PMMA bone cement on surrounding tissues was elucidated.

## Material and method

### Experimental animals and materials

SD rats (male, SPF grade, purchased from the Animal Experiment Cooperative Unit of the Third Hospital of Hebei Medical University, rat age:10 weeks, weight: 280 ± 20 g, certificate No. 20221004)(Code of Ethics Project, Medical Ethics Committee, The Third College of Hebei Medical College:z 2022-031-1), scanning electron microscope (SEM, Hebei Medical University Electron Microscopy Center, Hitachi, S-3500N), mobile C-arm X-ray machine (Siemens, Germany), Polymethyl methacrylate bone cement (Heraeus, Germany, 40 g/suit); enoxaparin sodium freeze-dried powder (Chengdu Baiyu Pharmaceutical Co., Ltd., 4,000 AxaIU/ branch, national medicine brand: H20150010); 3D printing standard size bone cement mold (Canghai 3D Printing Co., Ltd.), CCK-8 Kit (Item number:C0038, Shanghai Biyuntian Biotechnology Co., Ltd.), RAW264.7 cells (Item number: JH-M2084, Shanghai Jihe Biotechnology Co., Ltd.), paraffin slicer (model RM2135, Leica Germany), Optical microscope (model BX43, Japanese OLYMPUS company) and imaging system (model UC90, Japanese OLYMPUS company).

### Preparation of bone cement samples

Using 3D printing technology, we fabricated a cylindrical photosensitive resin mold with dimensions of 4 mm in diameter, 3 mm in length, and 2 mm in thickness. In order to ensure consistency, we prepared bone cement samples in the controlled environment of an operating room, maintaining a constant temperature and humidity throughout the process. To prepare the ES-PMMA bone cement samples, we first ground the bulk drug into powder using 8000AxaIU ES freeze-dried powder. Next, we mixed the powder with 40 g of PMMA bone cement powder. We then poured the bone cement liquid monomer into the powder mixture and stirred it quickly to ensure complete mixing. (PMMA bone cement sample preparation: 40 g PMMA bone cement powder and 20 ml bone cement liquid monomer were fully mixed). Once the ES-PMMA bone cement reached the dough stage, it was quickly implanted into a cylindrical mold and extruded on both sides. After the cement solidified, the specimen was removed and its size was measured using a Vernier caliper. To ensure the specimen was of high quality, it was examined by a C-arm X-ray machine to detect any air bubbles or inconsistencies in density. Only specimens that met the criteria for uniformity, size, and absence of air bubbles were selected. The preparation method for the bone cement in the PMMA group remained unchanged. Afterwards, both groups of bone cement specimens were subjected to high-power scanning electron microscopy to compare their respective characteristics.

## In vivo study

### Experimental grouping, surgical implantation, and sample collection

**Fig. 1:**
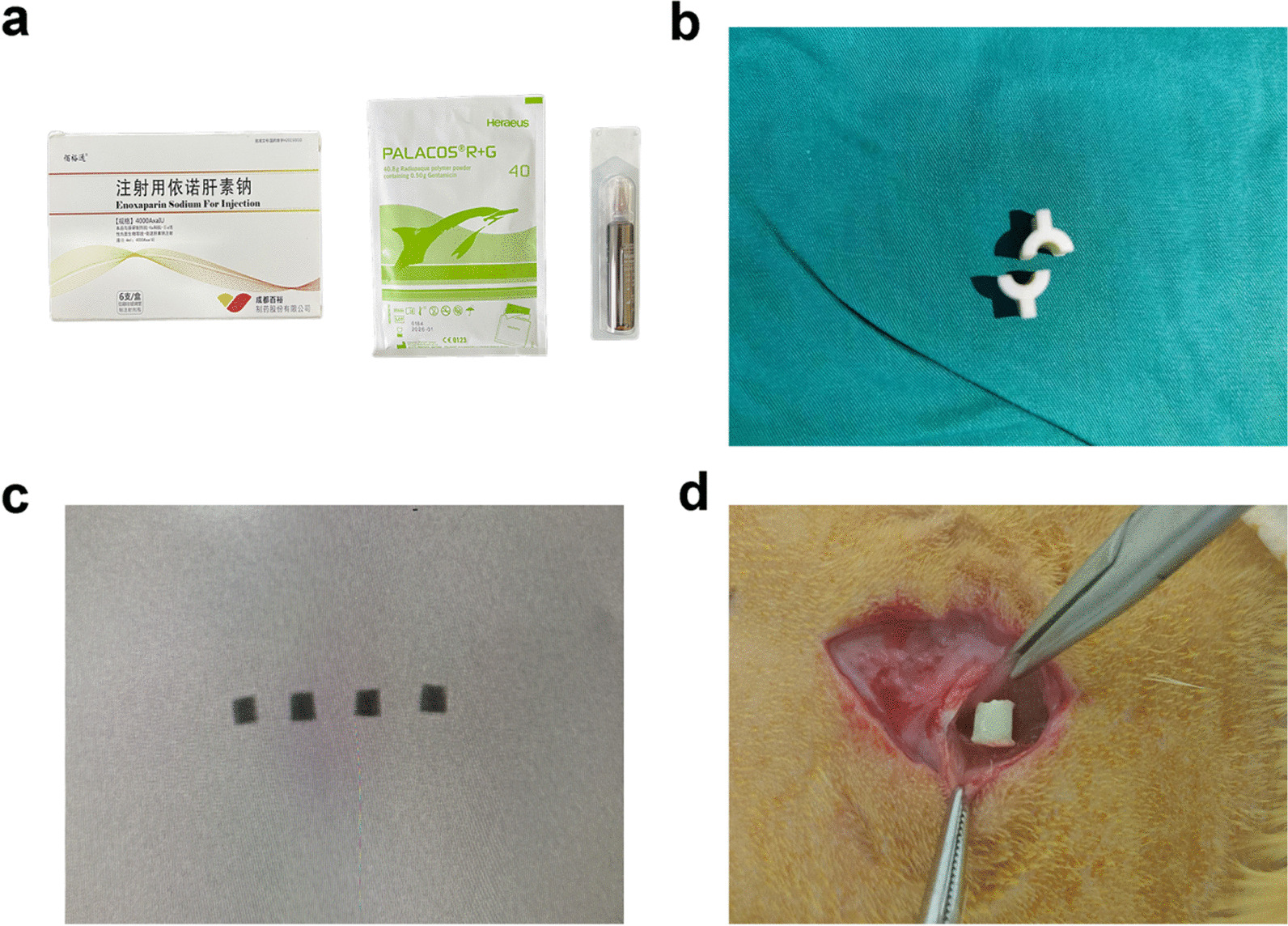
Related schematic diagram of in vitro experiment. **a** Enoxaparin sodium and PMMA bone cement. **b** Bone cement module with a length of 3 mm and a diameter of 4 mm. **c** Bone cement samples were examined with a C-arm X-ray machine. **d** Bone cement samples were surgically implanted

The procedures for using animals for surgical modeling were based on the guidelines for Laboratory Animal Welfare of Hebei Medical University and were approved by the Experimental Animal Ethics Committee of the Third Hospital of Hebei Medical University. The animals were raised under standard conditions and given one week to acclimate. In this study, a total of 36 male SD rats with an average weight of approximately 280 ± 20 g were utilized. The rats were randomly divided into two groups, with half of them receiving ES-PMMA bone cement samples and the other half receiving PMMA bone cement samples. Before the implantation, the dorsal area of each rat was shaved and sterilized. General anesthesia was administered using 3% sodium pentobarbital at a dosage of 50 mg/kg. The surgical procedure involved making an incision in the skin, then inserting a cement sample into the paravertebral muscle before closing the incision. Following the operation, the rats were housed in cages and provided with free access to food. To prevent infection, Penicillin G sodium 40,000 U was administered via injection into the thigh muscle of the rats for three consecutive days. After surgery, the animals were fed normally. Rats were sacrificed at 3, 7, and 14 days using anesthesia with an overdose of 3% sodium pentobarbital (100 mg/kg). Six rats per group were sacrificed at each time point. The bone cement and a small amount of surrounding tissue were removed from the same incision and stored in a 4% paraformaldehyde solution. Figure 1 depicts the related schematic diagram of the in vitro experiment.

### Immunofluorescence

The tissue paraffin sections underwent routine dewaxing to water before being placed in a high-pressure 3% citric acid repair solution to repair antigens. Next, 3% hydrogen peroxide was added for a 10-min period. Afterwards, primary antibodies (Anti-CD86 antibody and Anti-CD206 antibody) were dropped onto the sections at a 1:100 dilution and left overnight at 4℃. The slides underwent three rounds of washing with PBS buffer, each lasting 5 min. Subsequently, a fluorescent secondary antibody was applied in drops and incubated at 37 °C for 30 min. The slides were then washed again with PBS buffer for 5 min, repeated three times before drops of DAPI were applied, and incubated at room temperature for 10 min. The slides were rinsed with tap water and then sealed with a water-soluble sealant. Next, the slices were examined under a microscope at 100×, 200×, and 400× magnification. For each mouse, three non-repetitive fields were chosen at 200× magnification. Nine images were randomly selected for each time point in each group, and semi-quantitative analysis was performed using Image pro plus 6.0 software. The fluorescence intensity was then calculated.

### Immunohistochemical staining

The paraffin sections were first dewaxed in water. Antigen repair was carried out using 0.01 mol/L citrate buffer, and endogenous peroxidase activity was blocked using a 3% hydrogen peroxide solution. Primary antibody dilutions of anti-IL-6 (1:100), anti-TNF-α (1:100), and anti-IL-10 (1:100) were added and left to incubate overnight at 4 ℃. The slides underwent three rounds of rinsing with PBS for three minutes each. Following this, secondary antibodies were introduced and left to incubate at room temperature for 10–15 min. Two more rounds of PBS rinsing for three minutes each were conducted before staining with DAB. The next steps included hematoxylin counterstaining, dehydration, transparency, and sealing, followed by microscopic examination. The slices were observed under a light microscope at magnifications of 100×, 200×, and 400×. The cytoplasm of the positively stained cells appeared brown. Using Image pro plus 6.0 software, we analyzed the staining images and determined the expression of target proteins based on the depth of staining and distribution area. The resulting data was then plotted into bar charts using GraphPad Prism9 software.

## In vitro study

### Cell culture

The RAW264.7 cells were incubated in a complete medium consisting of DMEM medium containing 10% fetal bovine serum, as well as 100 U/mL penicillin and 100 mg/mL streptomycin. This incubation took place in a 5% CO_2_ incubator at a temperature of 37 °C.

### Preparation of conditioned medium

In this study, we weighed ES-PMMA and PMMA bone cement samples and then immersed them in the DMEM. Each gram of bone cement was added to 5 mL DMEM at 37 °C for 72 h. Afterward, we collected the immersion solution and centrifuged it at 5000 rpm for 10 min. We then collected the supernatant and filtered it through a 0.22 μm filter to obtain the bone cement extract with a concentration of 200 mg/mL. In order to create different concentrations of extract, the original extract was diluted with a complete medium resulting in four different concentrations: 25 mg/mL, 50 mg/mL, 75 mg/mL, and 100 mg/mL. Similarly, 4000 axAIU ES was fully dissolved in 1 ml of DMEM and then diluted with a complete medium to generate four different concentrations of ES: 100AxaIU/mL, 200AxaIU/mL, 300AxaIU/mL, and 400 AxaIU/mL.

### Cell proliferation activity

Cell Counting Kit8 (CCK8) was used to determine the cell proliferation [18, 19]. RAW264.7 cells were seeded into 96-well plates at a density of 5 × 10^3^ cells per well and incubated at 37 °C with 5% CO_2_ for around 24 h. The cells were then divided into three groups: blank, control, and experimental groups (ES, PMMA, and ES-PMMA). The RAW264.7 cells in the experimental groups were cultured with their corresponding conditioned medium. In the control group, RAW264.7 cells were cultured with a complete medium, while the blank group contained only the complete medium without cells. After 24 h, the CCK8 reagent was added and incubated for an additional 4 h. The absorbance at 450 nm was then measured on a microplate reader for each group, which was repeated three times. The relative cell viability was calculated using the following formula:

$$\begin{aligned} {\text{Cell proliferation activity}}\;(\%) & = \left( {{\text{OD}}_{{{\text{experimental}}}} - {\text{OD}}_{{{\text{blank}}}} } \right) \\ & \quad /\left( {{\text{OD}}_{{{\text{control}}}} - {\text{OD}}_{{{\text{blank}}}} } \right) \times 100\% . \\ \end{aligned}$$

### Cell treatment

The RAW264.7 cells were cultured in a complete medium at 37℃ and 5% CO2 for 24 h. Afterwards, they were incubated with 500 ng/mL LPS for an additional 24 h. The cells were then divided into five groups: the NC group (cells without LPS treatment), the LPS group, the LPS + ES group, the LPS + PMMA group, and the LPS + ES-PMMA group. After 24 h, the NC and LPS groups were switched to complete medium and cultured for an additional 24 h. Meanwhile, the LPS + ES, LPS + PMMA, and LPS + ES-PMMA groups were cultured with their respective conditioned medium for another 24 h.

### RT-qPCR

RT-qPCR was used to detect the expression of inflammatory factors in each group [20, 21]. Cells were collected, total RNA was extracted from cells using TRIzol reagent, and cDNA was synthesized by operating according to the reverse transcription kit instructions (SureScript First-Strand cDNA Synthesis Kit). RT-qPCR experiments were performed using cDNA as a template to detect the expression levels of TNF-α, IL-6, iNOS, IL-10, and Arg-1. 2 × SYBR Green qPCR MasterMix was used for RT–qPCR on a Bio-Rad iQ5 Real-Time PCR instrument, and GAPDH was used as the reference gene. The reaction conditions were set as follows: 1 cycle at 95 °C for 10 min; 40 cycles at 95 °C for 15 s, 55 °C for 30 s, and 72 °C for 30 s. All RT-qPCR samples were repeated three times, the average cycle threshold (CT) value was taken, and the relative mRNA expression levels of the above genes in each group were calculated by the 2-ΔΔCT method. The primer sequences were designed and synthesized by Wuhan Jinkaruo Biological Engineering Co., Ltd. The specific primer sequences are shown in Table 1.

**Table 1:** The sequences of the primers

Name	ACCESSION	Product lengths	Tm	Primer sequence
iNOS	NM_001313922	95	60.82	F:5′-CAGCTGGGCTGTACAAACCTT-3′
			58.43	R:5′-CATTGGAAGTGAAGCGTTTCG-3′
IL-6	NM_001314054	142	59.51	F:5′-CTTCCATCCAGTTGCCTTCTTG-3′
			59.00	R:5′-AATTAAGCCTCCGACTTGTGAAG-3′
Arg-1	NM_007482	249	58.37	F:5′-AACACTCCCCTGACAACCA-3′
			55.69	R:5′-CATCACCTTGCCAATCCC-3′
IL-10	NM_010548	105	58.45	F:5′-GCTCTTACTGACTGGCATGAG-3′
			60.88	R:5′-CGCAGCTCTAGGAGCATGTG-3′
TNF-α	NM_013693	164	59.31	F:5′-TCAGTTCCATGGCCCAGAC-3′
			59.84	R:5′-GTTGTCTTTGAGATCCATGCCATT-3′
GAPDH	NM_008084	79	59.83	F:5′-CCGCATCTTCTTGTGCAGTG-3′
			59.42	R:5′-CGATACGGCCAAATCCGTTC-3′

### Flow cytometry

Flow cytometry was used to detect the expression of CD86 and CD206 in each group [22, 23]. The cells were collected, packaged at 1 × 10^6^ cells/tube, and centrifuged at 1000 rpm for 5 min. PBS was added and resuspended, centrifuged at 1000 rpm for 5 min, and repeated twice. FITC CD86, FITC CD206, and isotype control antibodies were added to each EP tube and incubated in a dark place at 4 ℃ for 30 min. After centrifugation at 1000 rpm for 5 min, the supernatant was discarded, resuspended in PBS, centrifuged at 1000 rpm for 5 min, and repeated twice to remove the unbound antibody. Flow cytometric data were acquired using a BD FACSCalibur, and data were analyzed with FlowJo software.

### Western blot

Western blotting was used to detect the expression of TLR4/NF-κB signaling pathway-related proteins in each group [14, 20]. The cells were collected, lysed on ice for 30 min after washing with PBS, and centrifuged at 12 000 rpm for 15 min at 4 °C. After the determination of protein concentration by the BCA method, the appropriate amount of loading buffer was added to the protein samples and separated by electrophoresis on SDS polyacrylamide gel. Then the proteins on the gel were transferred to the PVDF membrane and blocked with 5% skim milk powder at room temperature for 2 h. Primary Anti-TLR4 antibody, Anti-p-NF-κB p65 antibody, and Anti-NF-κB p65 antibody (1: 1000) were added at 4 ℃ overnight, respectively. After washing the membrane, the corresponding secondary antibodies were added and incubated at room temperature for 2 h. After washing the film again and reacting with the luminescent reagent, the film was immediately placed in the exposure box, and the photosensitive film was exposed in a dark room for 1 min, and then developed and fixed. The films were scanned and imaged with an Epson Perfection V39 scanner. The gray value of the developed protein bands was measured by ImageJ software, and the relative protein expression was calculated using GAPDH as an internal reference.

### Statistical analysis

Statistical analysis was performed using GraphPad Prism 9. All experimental data were expressed as mean ± standard deviation (mean ± SD). Comparisons between two groups were formulated by t-test, whereas comparisons among multiple groups were assessed by one-way analysis of variance followed by Tukey’s multiple comparisons test. *P* < 0.05 means that the difference was statistically significant.

## Result

### Electron microscopic characterization of PMMA and ES-PMMA bone cement samples

**Fig. 2:**
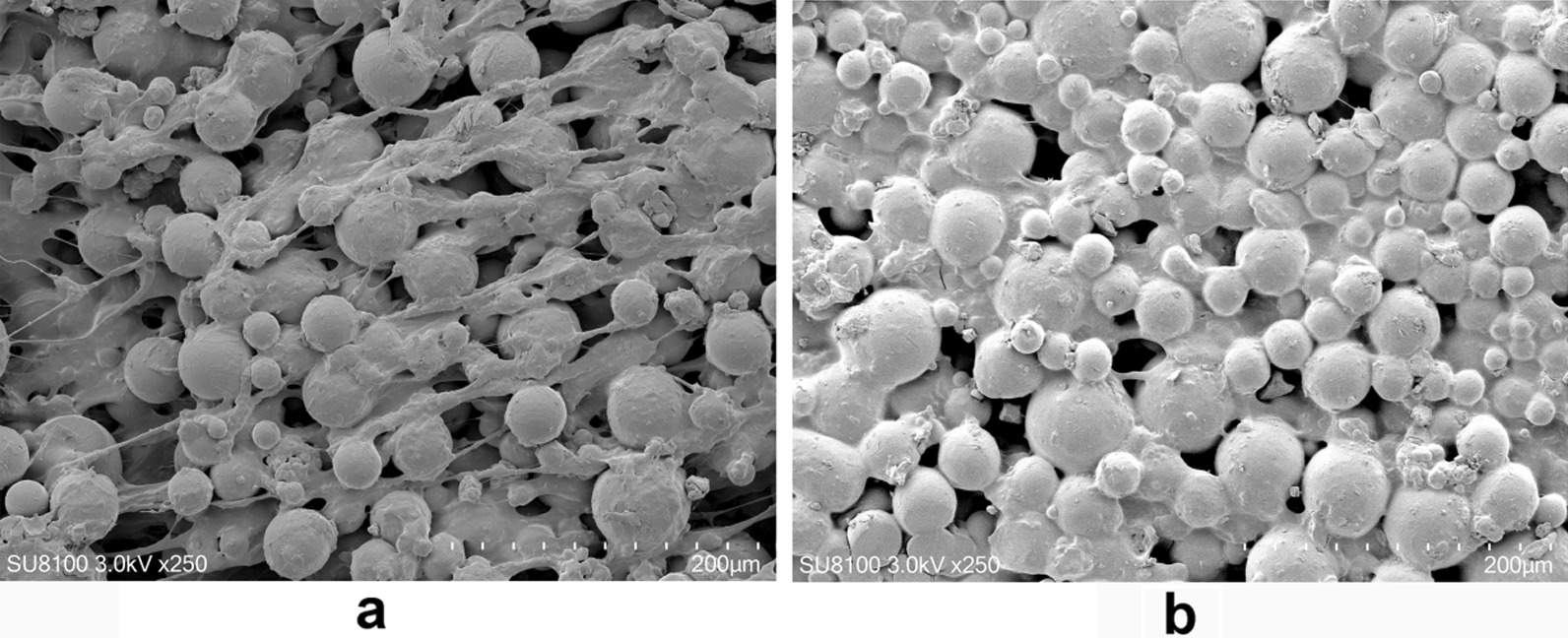
SEM images of PMMA and ES-PMMA bone cement. **a** PMMA. There are many voids between the cement particles. **b** ES-PMMA. Enoxaparin sodium was filled on the surface and space of bone cement particles

The bone cement samples from the PMMA group and ES-PMMA group were examined using a scanning electron microscope. It was evident that there were numerous pores present between the polymers of the PMMA group bone cement. In contrast, the ES-PMMA group exhibited sugar-coated substances that filled the surface of the bone cement and the spaces between the particles. We think that this syrup-like substance is enoxaparin, as shown in Fig. 2.

### Effect of ES-PMMA bone cement on macrophage polarization and expression of related inflammatory factors in surrounding tissues

**Fig. 3:**
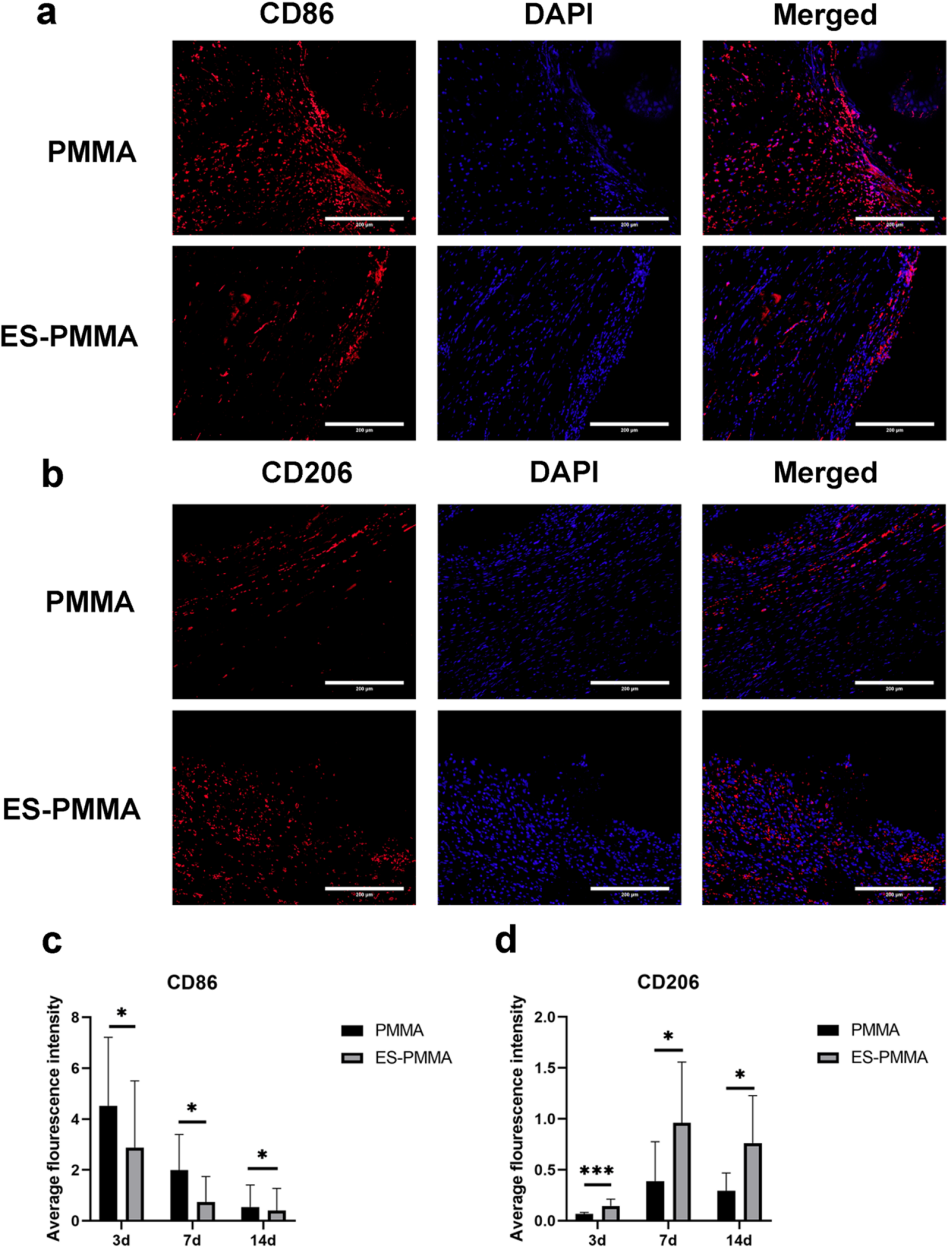
Expression and localization of CD86 and CD206 in bone cement surrounding tissues. The staining images were observed under a light microscope at ×200 magnification, red is the target protein, and the nucleus is blue. **a**, **b** are representative images of CD86 and CD206 in the two groups 7 days after surgery. **c** and** d** are the quantitative analysis of CD86 and CD206 in the tissue around bone cement (Mean ± SD. *n* = 6.* *P* ≤ 0.05; ** *P* ≤ 0.01; And ***P ≤ 0.001. Pictures taken 3 and 14 days after surgery are presented in Additional file 1)

**Fig. 4:**
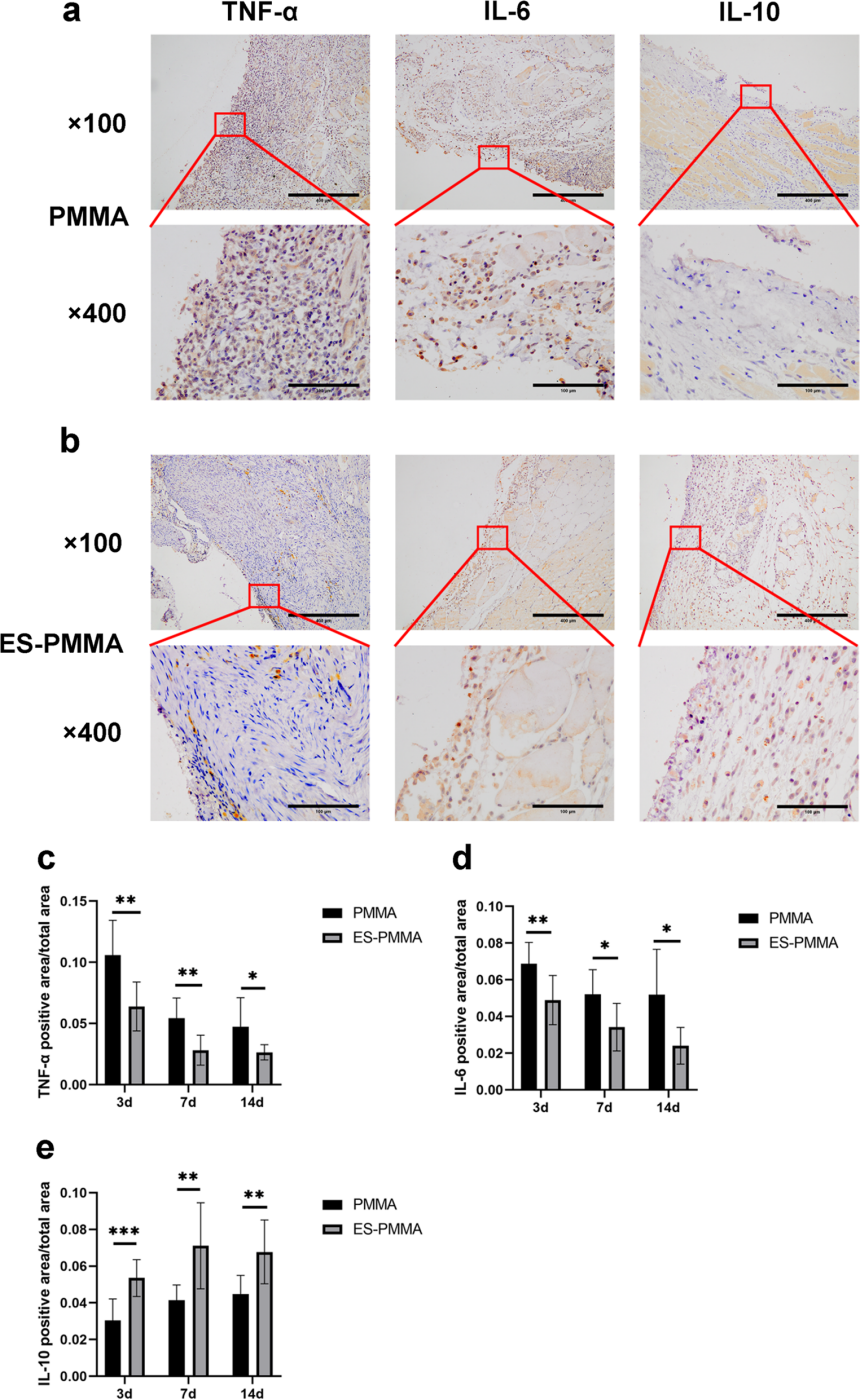
Expression of IL-6, IL-10, and TNF-α in the tissues surrounding bone cement. The staining images were observed under a light microscope at ×100 and ×400 magnification, and the brown-yellow color was the target protein. **a,** **b** were representative images of IL-6, IL-10, and TNF-α in the two groups 7 days after operation. **c**, **d**, and **e** are quantitative analyses of IL-6, IL-10, and TNF-α, respectively (Mean ± SD. *n* = 6. *P ≤ 0.05; **P ≤ 0.01; And ***P ≤ 0.001. Pictures taken 3 and 14 days after surgery are presented in Additional file 1)

The immunofluorescence results indicate that the expression level of the M2-specific marker CD206 was significantly higher in the ES-PMMA group, while the expression level of the M1-specific marker CD86 was lower compared to the PMMA group (Fig. 3). According to the results of the immunohistochemistry analysis, the ES-PMMA group displayed lower levels of positive expression for IL-6 and TNF-α compared to the PMMA group. Conversely, the positive expression level of IL-10 was higher in the ES-PMMA group than in the PMMA group (refer to Fig. 4).

### Screening of conditioned media

**Fig. 5:**
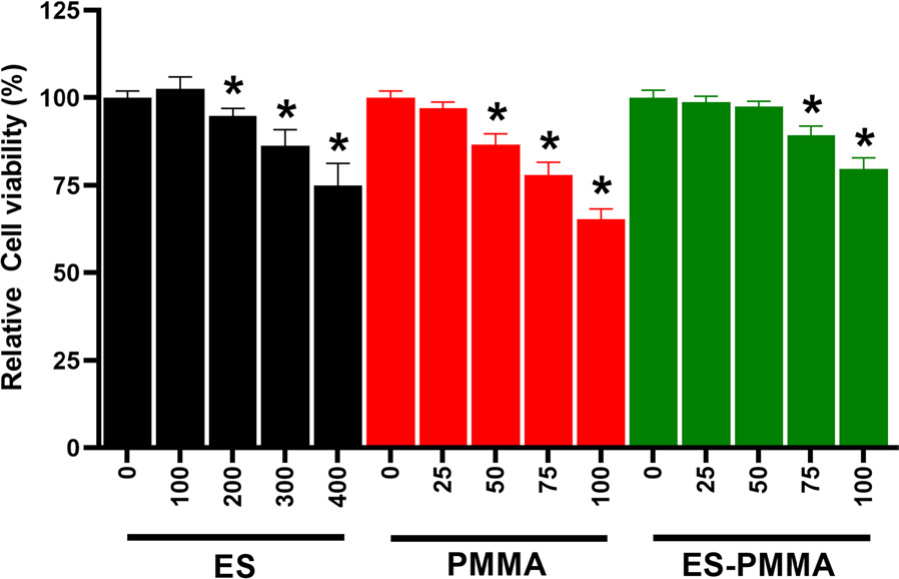
Screening of conditioned medium by CCK-8 assay: when the concentration of ES was 100AxaIU/ml and the two kinds of bone cement extracts were 25 mg/mL, the activity of RAW264.7 cells was not significantly affected (mean ± SD. *n* = 3. *P < 0.05 compared with control groups for each group)

Based on the results of the cell viability experiment, it was found that the concentration of ES 100AxaIU/ml and the two types of bone cement extracts at 25 mg/mL did not have any impact on the viability of RAW264.7 cells. As a result, these concentrations of conditioned medium were utilized to treat RAW264.7 cells in the subsequent experiments, as shown in Fig. 5.

### Effects of ES-PMMA bone cement on the polarization of RAW264.7 cells and the expression of related inflammatory factors

**Fig. 6:**
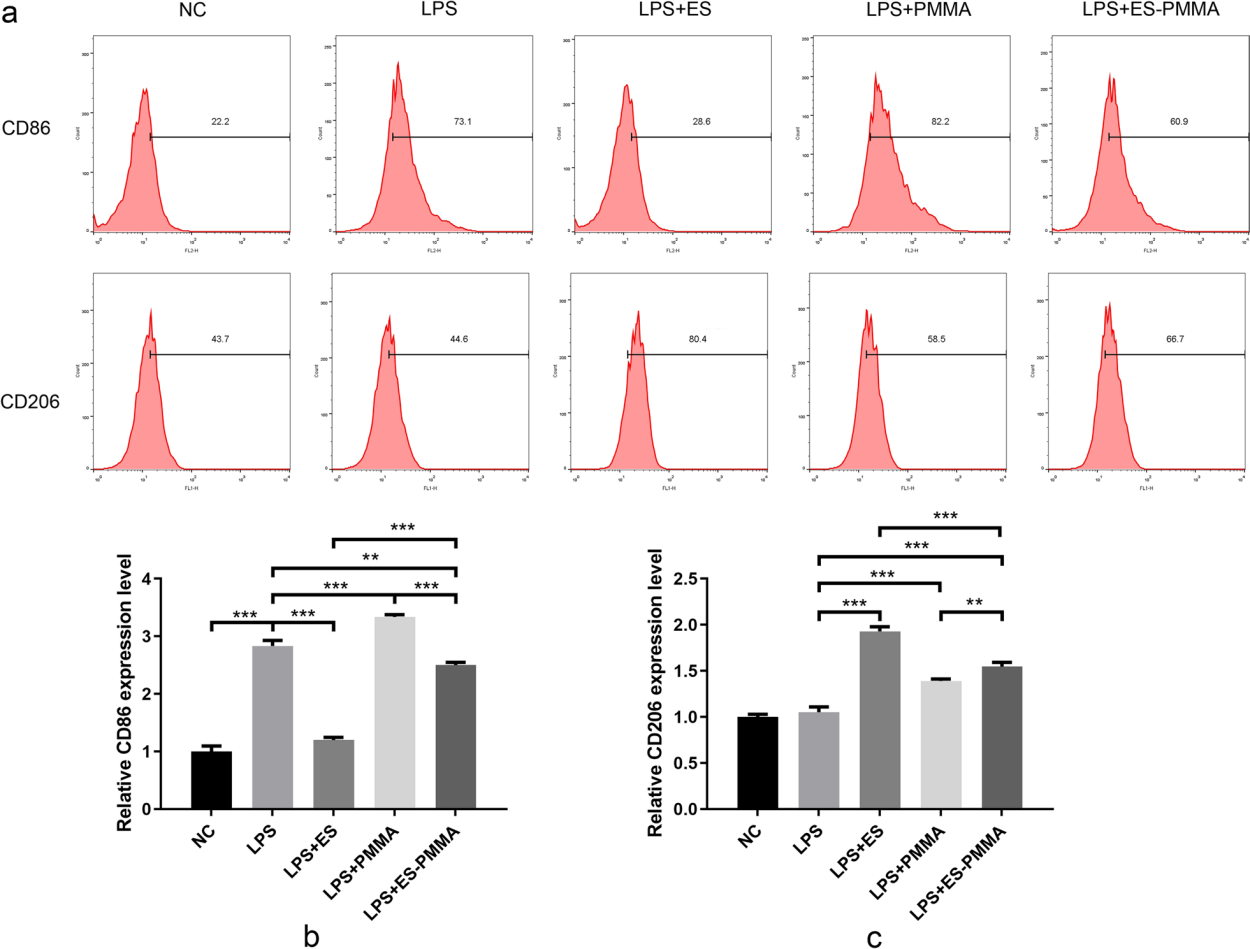
ES-PMMA bone cement induced polarization of macrophages to M2 phenotype. **a** Detection of CD86 and CD206 positive expression by flow cytometry. **b**, **c** Quantitative results of flow cytometry analysis.(Mean ± SD. *n* = 3. *P ≤ 0.05; **P ≤ 0.01; And ***P ≤ 0.001)

**Fig. 7:**
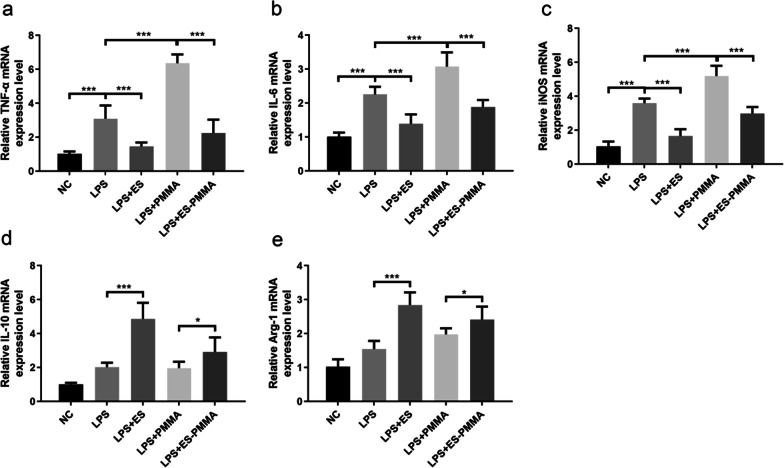
ES-PMMA bone cement decreased the levels of pro-inflammatory factors and increased the levels of anti-inflammatory factors. **a–e** is the expression of TNF-α, IL-6,iNOS,IL-10 and Arg-1 in each group (Mean ± SD. n = 3. *P ≤ 0.05; **P ≤ 0.01; And ***P ≤ 0.001)

In comparison to the NC group, the LPS group showed an increase in the expression of the M1 macrophage marker CD86, as well as M1 macrophage-related proinflammatory cytokines TNF-α, IL-6, and iNOS. This provides evidence that the inflammatory model of RAW264.7 cells induced by LPS was successfully established. The LPS + ES group demonstrated a decrease in the expression levels of CD86, TNF-α, IL-6, and iNOS, while showing an increase in the expression levels of the M2 macrophage marker CD206 and M2 macrophage-related anti-inflammatory cytokines (IL-10, Arg-1) when compared to the LPS group. Similarly, the LPS + ES-PMMA group showed a reduction in the expression levels of CD86, TNF-α, IL-6, and iNOS, and an increase in the expression levels of CD206, IL-10, and Arg-1, when compared to the LPS + PMMA group (Figs. 6, 7).

### Effect of ES-PMMA bone cement on TLR4/NF-κB signaling pathway

**Fig. 8:**
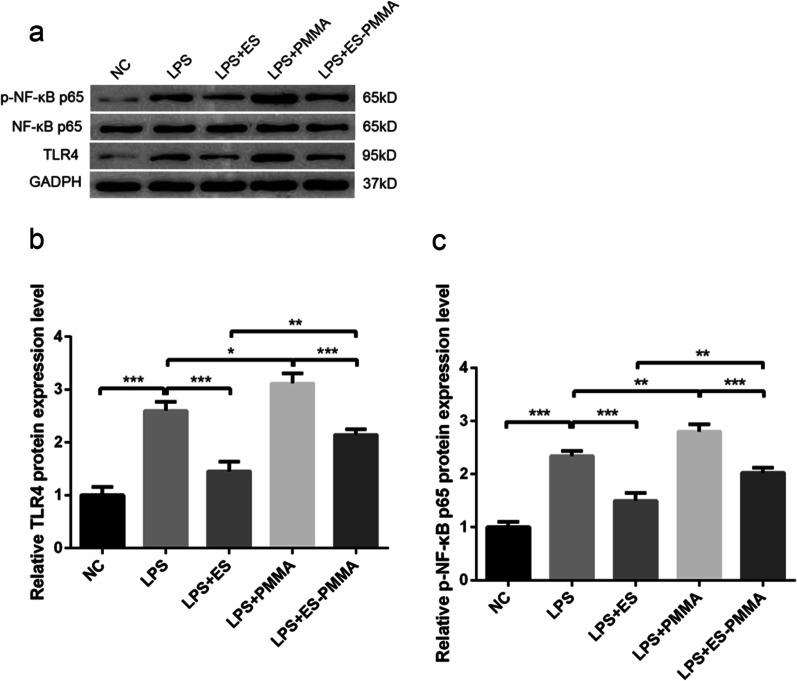
ES-PMMA bone cement inhibited TLR4 expression and NF-κB phosphorylation compared with PMMA bone cement. **a** The protein expression levels of rats in each group were determined by Western blotting. **b** TLR4/GADPH expression levels in each group. **c** The expression level of p-NF-κB p65/ NF-κB p65 in each group (Mean ± SD. *n* = 3. *P ≤ 0.05; **P ≤ 0.01; And ***P ≤ 0.001)

The levels of TLR4/GAPDH and p-NF-κB p65/NF-κB p65 were significantly reduced in the LPS + ES group when compared to the LPS group. Additionally, the LPS + ES-PMMA group showed a decrease in these levels when compared to the LPS + PMMA group. These findings suggest that LPS + ES-PMMA inhibits TLR4 expression and NF-κB phosphorylation more effectively than the LPS + PMMA group (Fig. 8).

## Discussion

Enoxaparin sodium has been reported to possess both anticoagulant and anti-inflammatory properties [24–26]. Building on the success of antibiotic-loaded bone cement, PMMA bone cement can also serve as a suitable drug carrier [27, 28]. In their study, Sun et al. [13] demonstrated that the use of bone cement loaded with varying amounts of enoxaparin sodium effectively released the substance. Furthermore, the released enoxaparin sodium was found to have a local anticoagulant effect. This highlights the potential of ES-PMMA bone cement as a viable option for the controlled release of enoxaparin sodium. Sang et al. [14] conducted an in vitro study using New Zealand rabbits to establish a carotid shunt model. Their research compared PMMA bone cement with ES-PMMA bone cement and concluded that ES-PMMA bone cement is effective in reducing local thrombosis. This is achieved by reducing the expression of the thrombosis-related regulatory protein CD40 in vascular endothelial cells. The study findings suggest that ES-PMMA can release pharmaceutically active enoxaparin sodium. Overall, these two experiments provide evidence for the potential of ES-PMMA as a treatment option.

When bone cement is implanted in the body, macrophages quickly arrive at the injury site and produce inflammatory cytokines and chemokines. They continue to perform various functions until tissue repair is finished [29, 30]. To adjust to the surrounding microenvironment during tissue repair, macrophages are typically categorized into M1 and M2 types. M1-type macrophages are known for their exceptional phagocytic abilities, allowing them to efficiently eliminate invading pathogens and initiate an inflammatory response to protect the host. While they are crucial in the early stages of inflammation, an excessive amount of macrophages can hinder tissue regeneration, disrupt wound healing, and ultimately harm the host [31]. M2 macrophages play a crucial role in inhibiting inflammatory responses, removing debris and apoptotic cells, promoting tissue repair and wound healing, and enhancing immune regulation [32, 33]. However, macrophages can also be polarized into M1-type macrophages under the stimulation of LPS and/or IFN-γ. These M1 macrophages have specific markers such as CD80 and CD86 on their cell surface and can produce a significant amount of TNF-α, IL-6, and IL-1β proinflammatory factors, as well as iNOS [34]. M2 macrophages are primarily stimulated and differentiated by IL-4 and IL-13. These cytokines are responsible for the expression of Arg-1 and CD206, as well as the secretion of anti-inflammatory cytokines like IL-10 and IL-13 [35]. In this study, the LPS + ES-PMMA group showed a significant decrease in the expression levels of CD86, TNF-α, IL-6, and iNOS, while exhibiting an increase in the expression levels of CD206, IL-10, and Arg-1 when compared to the LPS + PMMA group. Additionally, the LPS + ES group exhibited a decrease in pro-inflammatory factors and an increase in anti-inflammatory factors when compared to the LPS group. In comparison to PMMA bone cement, ES-PMMA bone cement has been found to induce M2 polarization of macrophages and increase the secretion of anti-inflammatory factors. This effect may be attributed to the release of ES. HMGB-1, a nuclear protein, has been shown to have a highly proinflammatory effect when released into the intercellular space [36]. In their study, Nadine A. Kerr et al. [37] demonstrated that enoxaparin sodium has the ability to target HMGB1 and alleviate the inflammatory response in individuals with traumatic brain injury. This treatment also effectively reduces the release of inflammatory factors. Monocytes can be recruited and transformed into proinflammatory macrophages through the influence of GM-CSF [38]. According to Lean et al. [25], enoxaparin treatment can reduce GM-CSF levels, leading to a decrease in the number of M1 macrophages and an increase in the number of M2 macrophages. In their research, Litov et al. [15] demonstrated that LMWH exhibits a strong affinity for IFNγ and, at a certain concentration, can effectively obstruct its interaction with the cellular receptor. This results in the inhibition of the IFNγ signaling pathway and the expression of IFNγ-induced proteins, which serves as a molecular mechanism for anti-inflammatory effects. These findings suggest that LMWH possesses anti-inflammatory properties. In this study's animal experiments, the results of immunohistochemistry and immunofluorescence indicate that the tissues surrounding ES-PMMA bone cement had a higher proportion of CD206-positive cells, a marker for M2 macrophages, and increased positive expression of the M2 macrophage-related cytokine IL-10 compared to the PMMA group. This suggests that ES-PMMA bone cement can still play an anti-inflammatory role by releasing ES locally in the tissue, which is consistent with the findings of Rebecca Lever et al. The study by Lever et al. [39] showed that topical application of heparin in vivo model of peritonitis in rats can also exert anti-inflammatory effects, which provides the possibility of topical application of low molecular weight heparin.

Studies have shown that [40–44] macrophage polarization is closely related to TLR4/NF-κB signaling pathway. TLRs bind to ligands and act on MyD88, which promotes the phosphorylation of downstream IL-1R-related kinases and recruitment of TRAF6 to the cell membrane. TRAF6 enriches the TAK complex, thereby regulating the activity of Iκ-B subunits. However, it affects the activation of NF-κB transcription factors in inactive macrophages. NF-κB is a key transcriptional regulator of TLR4-induced M1 macrophage polarization, so in most cases, TLR4/NF-κB signaling converts macrophages to the M1 phenotype. Research has demonstrated [45] that glioma exosomes have the ability to hinder the NF-κB pathway while simultaneously encouraging M2 macrophage polarization. Cigarran Guldris et al. [46] discovered that following the creation of the UUO model, M1 macrophages were the dominant type in renal tissue, and the expression of the TLR4/NF-κB signaling pathway was increased. However, the administration of TLR4 inhibitor TAK-242 resulted in a decrease in M1 macrophages and an increase in M2 macrophages. Suggesting that modulation of the NF-κB pathway can regulate the polarization of macrophages to the M2 phenotype. In this study, it was found that the levels of TLR4/GAPDH and p-NF-κB p65/NF-κB p65 were significantly lower in the LPS + ES group than in the LPS group. Additionally, the LPS + ES-PMMA group showed greater inhibition of TLR4 expression and NF-κB phosphorylation compared to the LPS + PMMA group, indicating a suppression of the TLR4/NF-κB signaling pathway. Therefore, we speculate that ES-PMMA bone cement can induce polarization of M2 macrophages by down-regulating TLR4/NF-κB signaling pathway. Wu et al. [16] demonstrated that LMWH has an effect on the TLR4-MyD88-NF-κB signaling pathway, leading to improvements in the inflammatory state of rats with acute sinusitis by inhibiting this pathway. There are two distinct inhibitory mechanisms associated with heparin on the NF-κB signaling pathway. The first mechanism involves inhibiting the translocation of the transcription factor into the nucleus, while the second mechanism is believed to involve heparin interfering with the ability of NF-κB to bind to DNA in the nucleus in a non-specific manner [47–49].

## Conclusions

In conclusion, compared with PMMA bone cement, ES-PMMA bone cement can more strongly down-regulate the expression of TLR4/NF-κB signaling pathway, induce macrophages to be polarized towards M2 phenotype, and play an important role in anti-inflammatory immune regulation. However, the specific regulatory process and mechanism of action have not been elucidated, and further studies are needed.

## Supplementary Information

**Additional file 1. 3d 14d:** Immunohistochemical results of PMMA group and ES-PMMA group at 3 and 14 days after surgery. **3d:** Immunofluorescence results of PMMA group and ES-PMMA group at 3 days after surgery. **14d:** Immunofluorescence results of PMMA group and ES-PMMA group at 14 days after surgery. **NF-κB p65 1:** The original blots/gels of NF-κB p65 (1). **NF-κB p65 2:** The original blots/gels of NF-κB p65 (2). **NF-κB p65 3:** The original blots/gels of NF-κB p65 (3). **NF-κB p65 1:** The original blots/gels of NF-κB p65 (1). **p-NF-κB p65 -1:** The original blots/gels of p-NF-κB p65 (1). **p-NF-κB p65 -2**: The original blots/gels of p-NF-κB p65 (2). **p-NF-κB p65 -3:** The original blots/gels of p-NF-κB p65 (3). **TLR4 1:** The original blots/gels of p-TLR4 (1). **TLR4 2:** The original blots/gels of p-TLR4 (2). **TLR4 3:** The original blots/gels of p-TLR4 (3).

## Data Availability

The datasets used and analyzed during the current study are available from the corresponding author upon reasonable request.

## Abbreviations

PMMA: Polymethyl methacrylate
ES: Enoxaparin sodium
SEM: Scanning electron microscopy
LPS: Lipopolysaccharide
UUO: Unilateral ureteral obstruction
GM-CSF: Granulocyte–macrophage colony-stimulating factor
