# Supplementary figures and images for: Enoxaparin sodium bone cement plays an anti-inflammatory immunomodulatory role by inducing the polarization of M2 macrophages
Source: J Orthop Surg Res. 2023 May 23;18:380. doi: 10.1186/s13018-023-03865-8 (PMC10207791; doi:10.1186/s13018-023-03865-8)

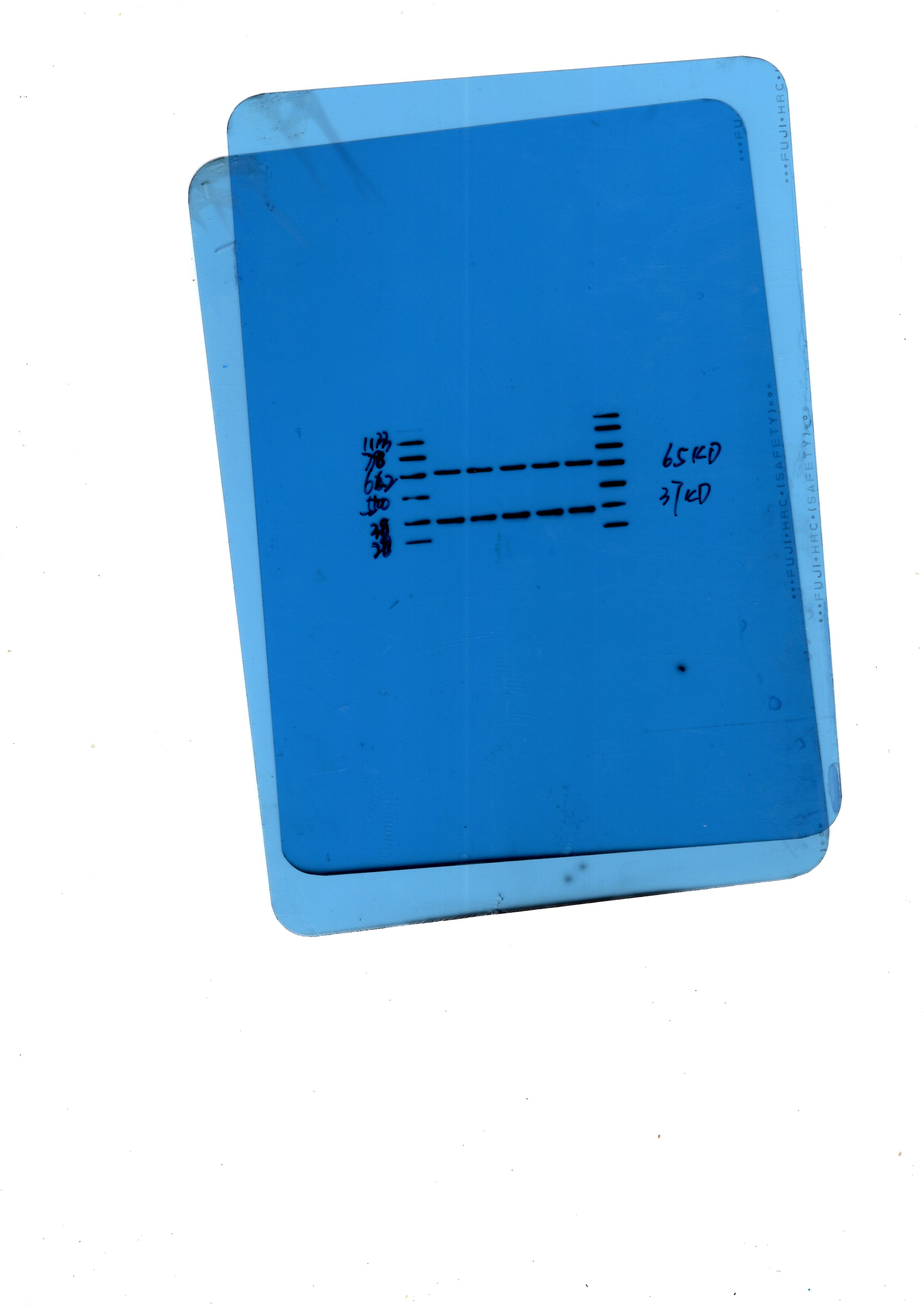

Supplement: Supplementary file 1 — Additional file 1. 3d 14d: Immunohistochemical results of PMMA group and ES-PMMA group at 3 and 14 days after surgery. 3d: Immunofluorescence results of PMMA group and ES-PMMA group at 3 days after surgery. 14d: Immunofluorescence results of PMMA group and ES-PMMA group at 14 days after surgery. NF-κB p65 1: The original blots/gels of NF-κB p65 (1). NF-κB p65 2: The original blots/gels of NF-κB p65 (2). NF-κB p65 3: The original blots/gels of NF-κB p65 (3). NF-κB p65 1: The original blots/gels of NF-κB p65 (1). p-NF-κB p65 -1: The original blots/gels of p-NF-κB p65 (1). p-NF-κB p65 -2: The original blots/gels of p-NF-κB p65 (2). p-NF-κB p65 -3: The original blots/gels of p-NF-κB p65 (3). TLR4 1: The original blots/gels of p-TLR4 (1). TLR4 2: The original blots/gels of p-TLR4 (2). TLR4 3: The original blots/gels of p-TLR4 (3). [file 13018_2023_3865_MOESM1_ESM.zip › Supplementary material/NF-a╩B p65 1.jpg]

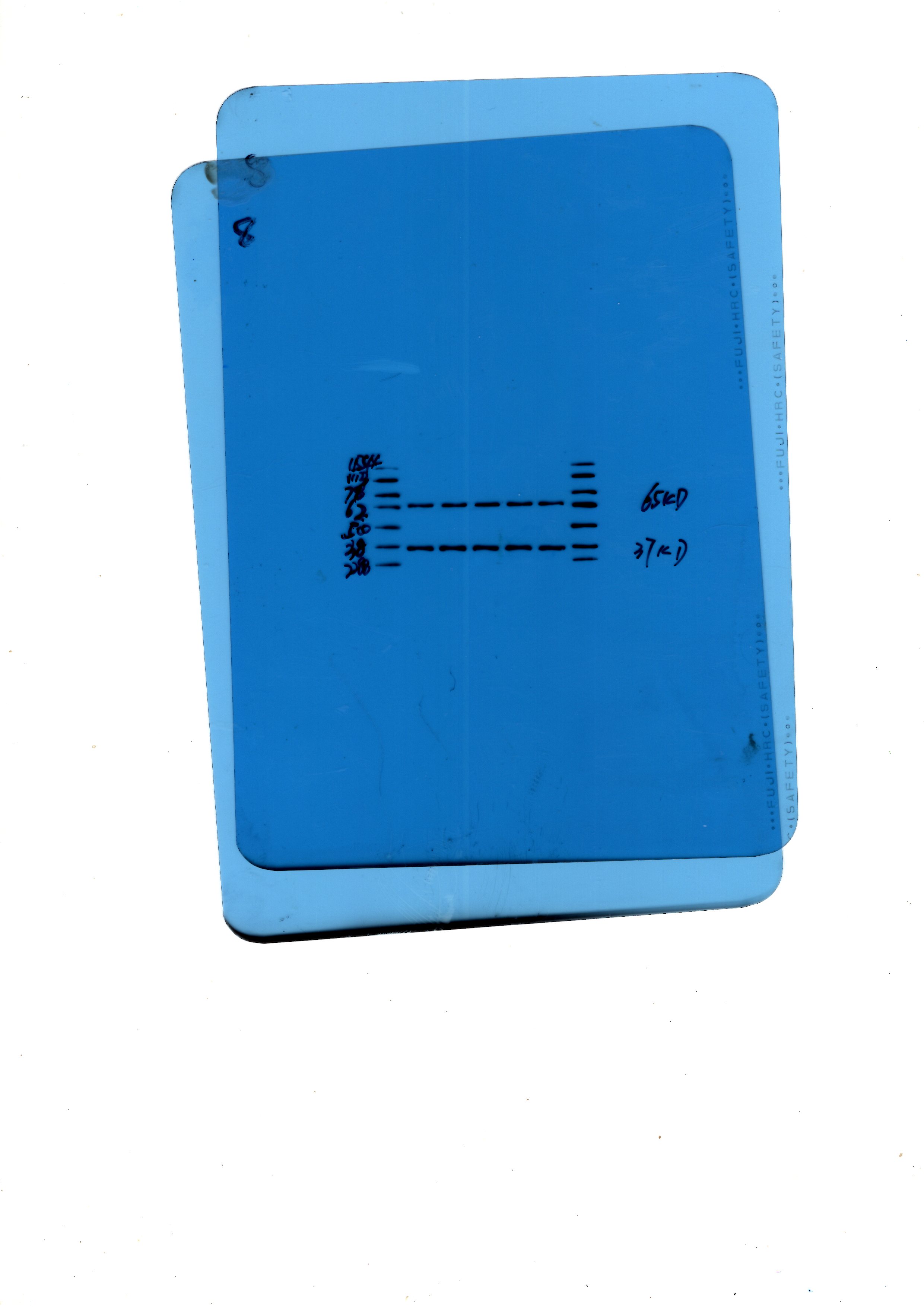

Supplement: Supplementary file 1 — Additional file 1. 3d 14d: Immunohistochemical results of PMMA group and ES-PMMA group at 3 and 14 days after surgery. 3d: Immunofluorescence results of PMMA group and ES-PMMA group at 3 days after surgery. 14d: Immunofluorescence results of PMMA group and ES-PMMA group at 14 days after surgery. NF-κB p65 1: The original blots/gels of NF-κB p65 (1). NF-κB p65 2: The original blots/gels of NF-κB p65 (2). NF-κB p65 3: The original blots/gels of NF-κB p65 (3). NF-κB p65 1: The original blots/gels of NF-κB p65 (1). p-NF-κB p65 -1: The original blots/gels of p-NF-κB p65 (1). p-NF-κB p65 -2: The original blots/gels of p-NF-κB p65 (2). p-NF-κB p65 -3: The original blots/gels of p-NF-κB p65 (3). TLR4 1: The original blots/gels of p-TLR4 (1). TLR4 2: The original blots/gels of p-TLR4 (2). TLR4 3: The original blots/gels of p-TLR4 (3). [file 13018_2023_3865_MOESM1_ESM.zip › Supplementary material/NF-a╩B p65 2.jpg]

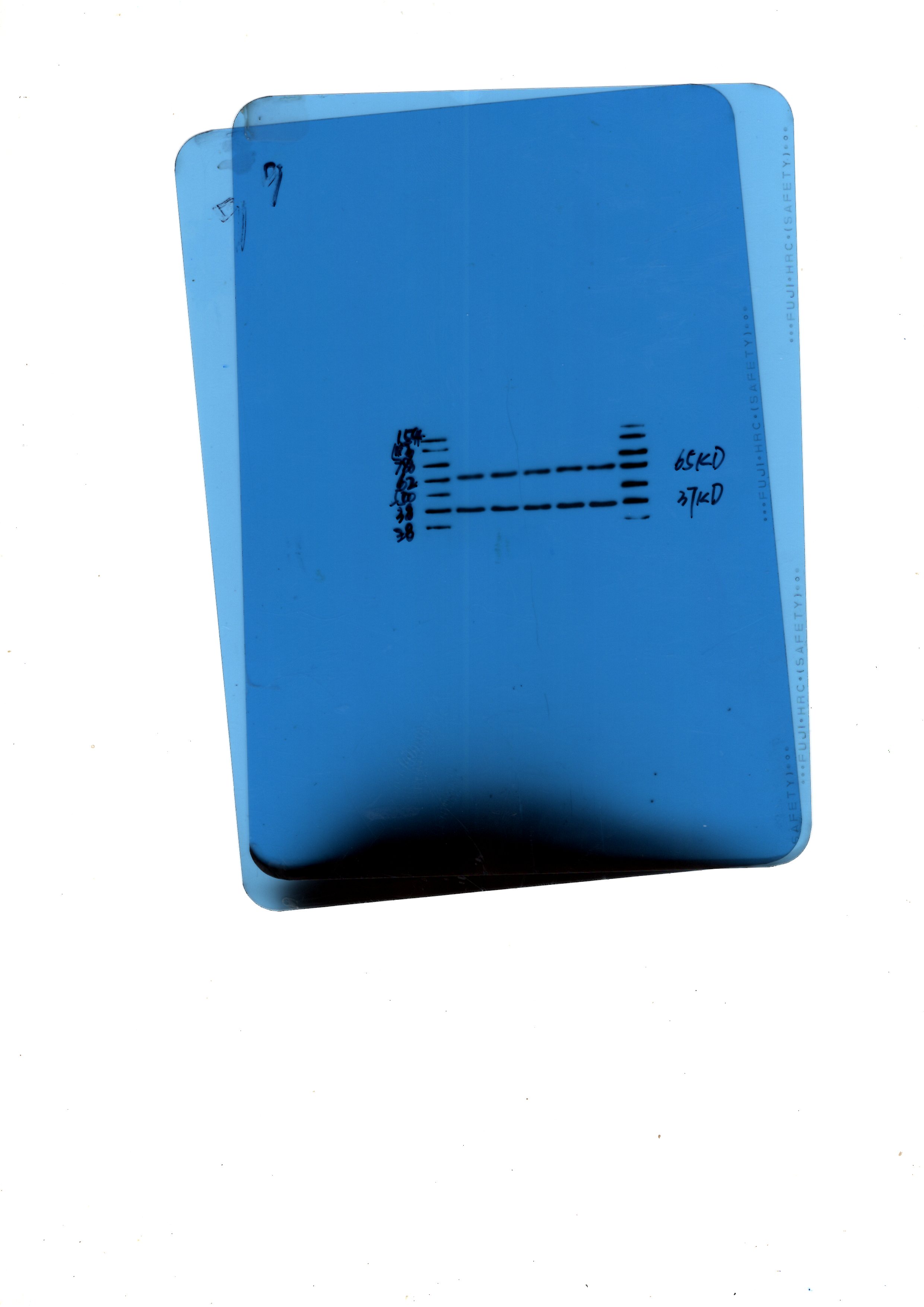

Supplement: Supplementary file 1 — Additional file 1. 3d 14d: Immunohistochemical results of PMMA group and ES-PMMA group at 3 and 14 days after surgery. 3d: Immunofluorescence results of PMMA group and ES-PMMA group at 3 days after surgery. 14d: Immunofluorescence results of PMMA group and ES-PMMA group at 14 days after surgery. NF-κB p65 1: The original blots/gels of NF-κB p65 (1). NF-κB p65 2: The original blots/gels of NF-κB p65 (2). NF-κB p65 3: The original blots/gels of NF-κB p65 (3). NF-κB p65 1: The original blots/gels of NF-κB p65 (1). p-NF-κB p65 -1: The original blots/gels of p-NF-κB p65 (1). p-NF-κB p65 -2: The original blots/gels of p-NF-κB p65 (2). p-NF-κB p65 -3: The original blots/gels of p-NF-κB p65 (3). TLR4 1: The original blots/gels of p-TLR4 (1). TLR4 2: The original blots/gels of p-TLR4 (2). TLR4 3: The original blots/gels of p-TLR4 (3). [file 13018_2023_3865_MOESM1_ESM.zip › Supplementary material/NF-a╩B p65 3.jpg]

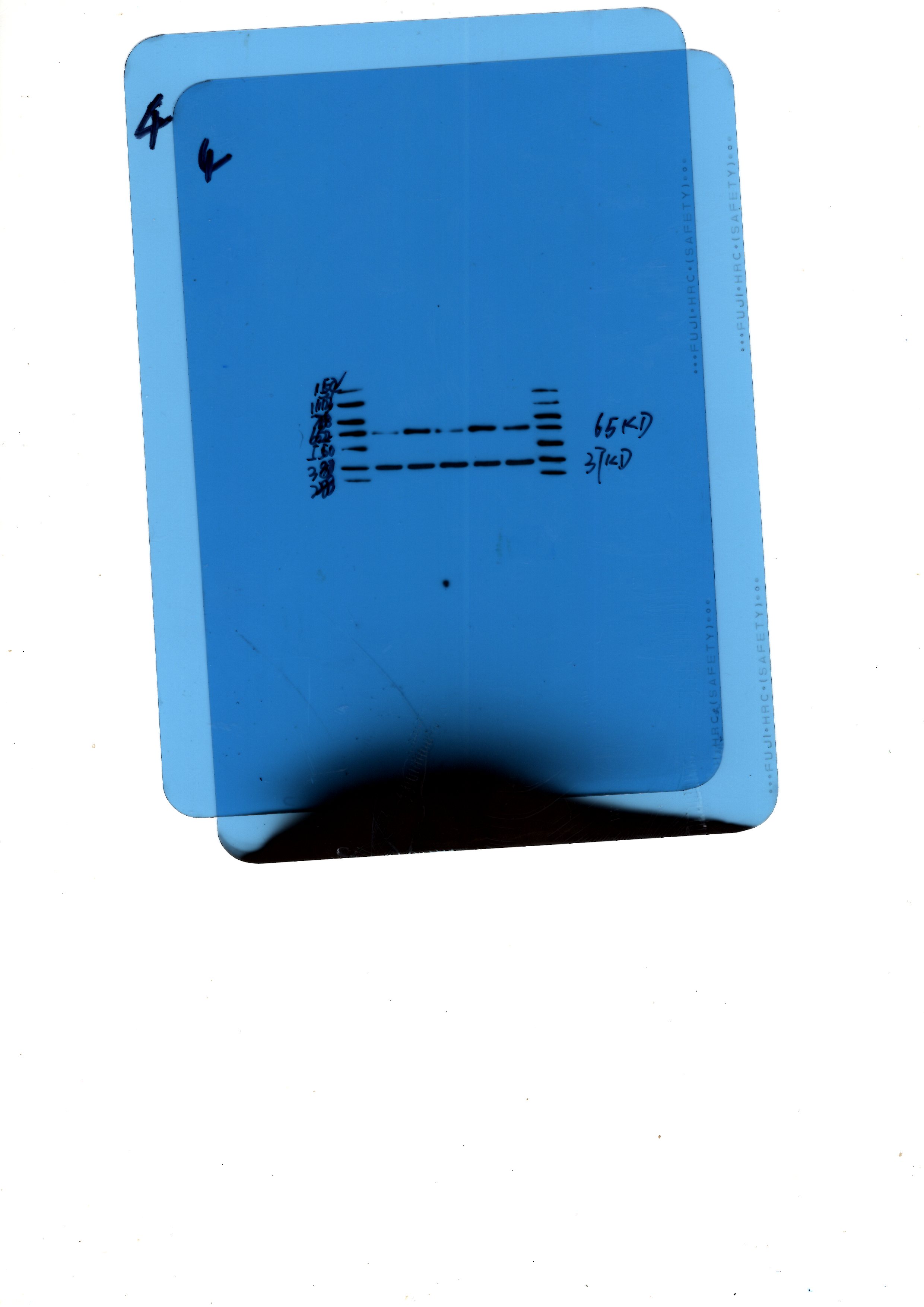

Supplement: Supplementary file 1 — Additional file 1. 3d 14d: Immunohistochemical results of PMMA group and ES-PMMA group at 3 and 14 days after surgery. 3d: Immunofluorescence results of PMMA group and ES-PMMA group at 3 days after surgery. 14d: Immunofluorescence results of PMMA group and ES-PMMA group at 14 days after surgery. NF-κB p65 1: The original blots/gels of NF-κB p65 (1). NF-κB p65 2: The original blots/gels of NF-κB p65 (2). NF-κB p65 3: The original blots/gels of NF-κB p65 (3). NF-κB p65 1: The original blots/gels of NF-κB p65 (1). p-NF-κB p65 -1: The original blots/gels of p-NF-κB p65 (1). p-NF-κB p65 -2: The original blots/gels of p-NF-κB p65 (2). p-NF-κB p65 -3: The original blots/gels of p-NF-κB p65 (3). TLR4 1: The original blots/gels of p-TLR4 (1). TLR4 2: The original blots/gels of p-TLR4 (2). TLR4 3: The original blots/gels of p-TLR4 (3). [file 13018_2023_3865_MOESM1_ESM.zip › Supplementary material/p-NF-a╩B p65 -1.jpg]

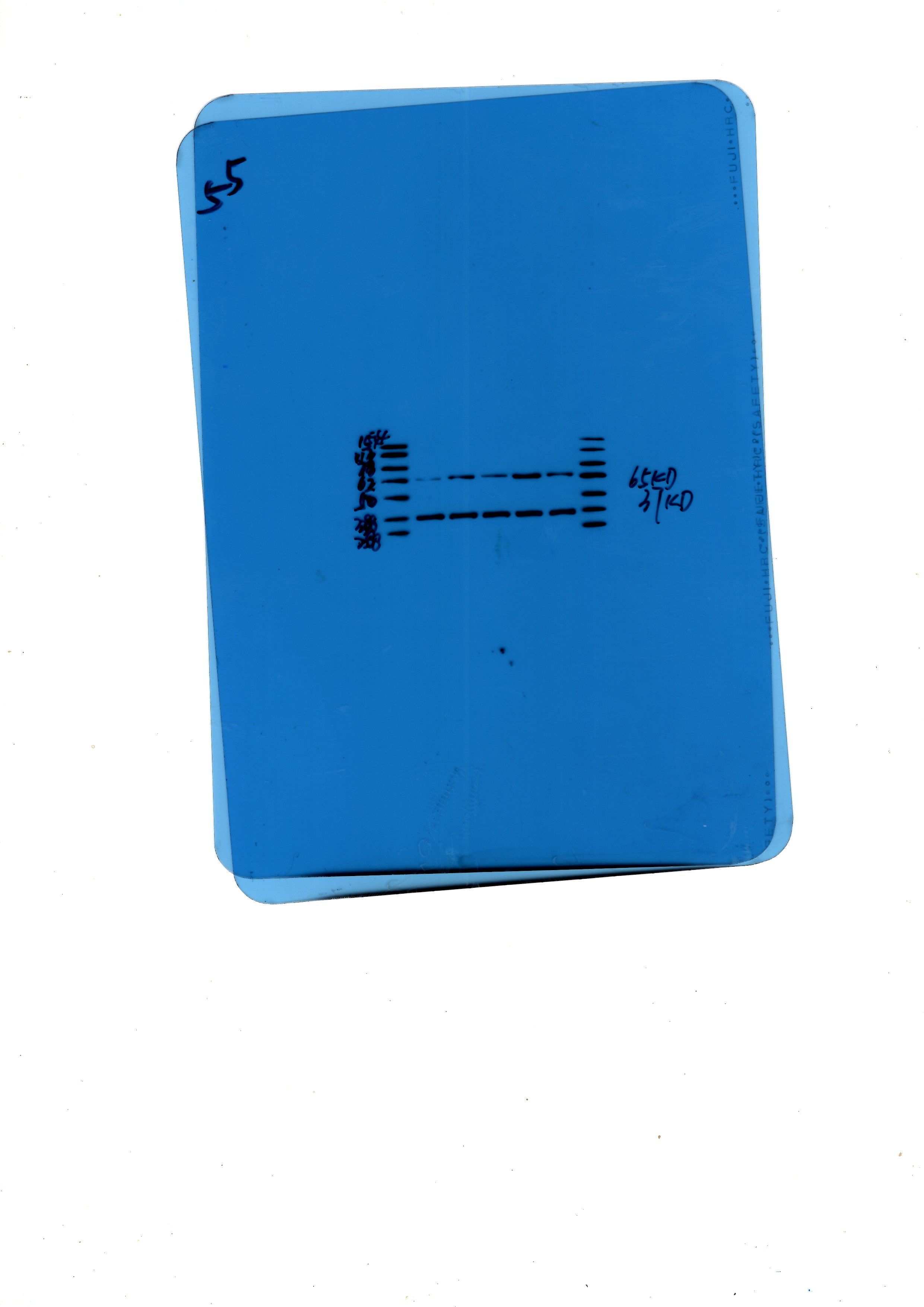

Supplement: Supplementary file 1 — Additional file 1. 3d 14d: Immunohistochemical results of PMMA group and ES-PMMA group at 3 and 14 days after surgery. 3d: Immunofluorescence results of PMMA group and ES-PMMA group at 3 days after surgery. 14d: Immunofluorescence results of PMMA group and ES-PMMA group at 14 days after surgery. NF-κB p65 1: The original blots/gels of NF-κB p65 (1). NF-κB p65 2: The original blots/gels of NF-κB p65 (2). NF-κB p65 3: The original blots/gels of NF-κB p65 (3). NF-κB p65 1: The original blots/gels of NF-κB p65 (1). p-NF-κB p65 -1: The original blots/gels of p-NF-κB p65 (1). p-NF-κB p65 -2: The original blots/gels of p-NF-κB p65 (2). p-NF-κB p65 -3: The original blots/gels of p-NF-κB p65 (3). TLR4 1: The original blots/gels of p-TLR4 (1). TLR4 2: The original blots/gels of p-TLR4 (2). TLR4 3: The original blots/gels of p-TLR4 (3). [file 13018_2023_3865_MOESM1_ESM.zip › Supplementary material/p-NF-a╩B p65 -2.jpg]

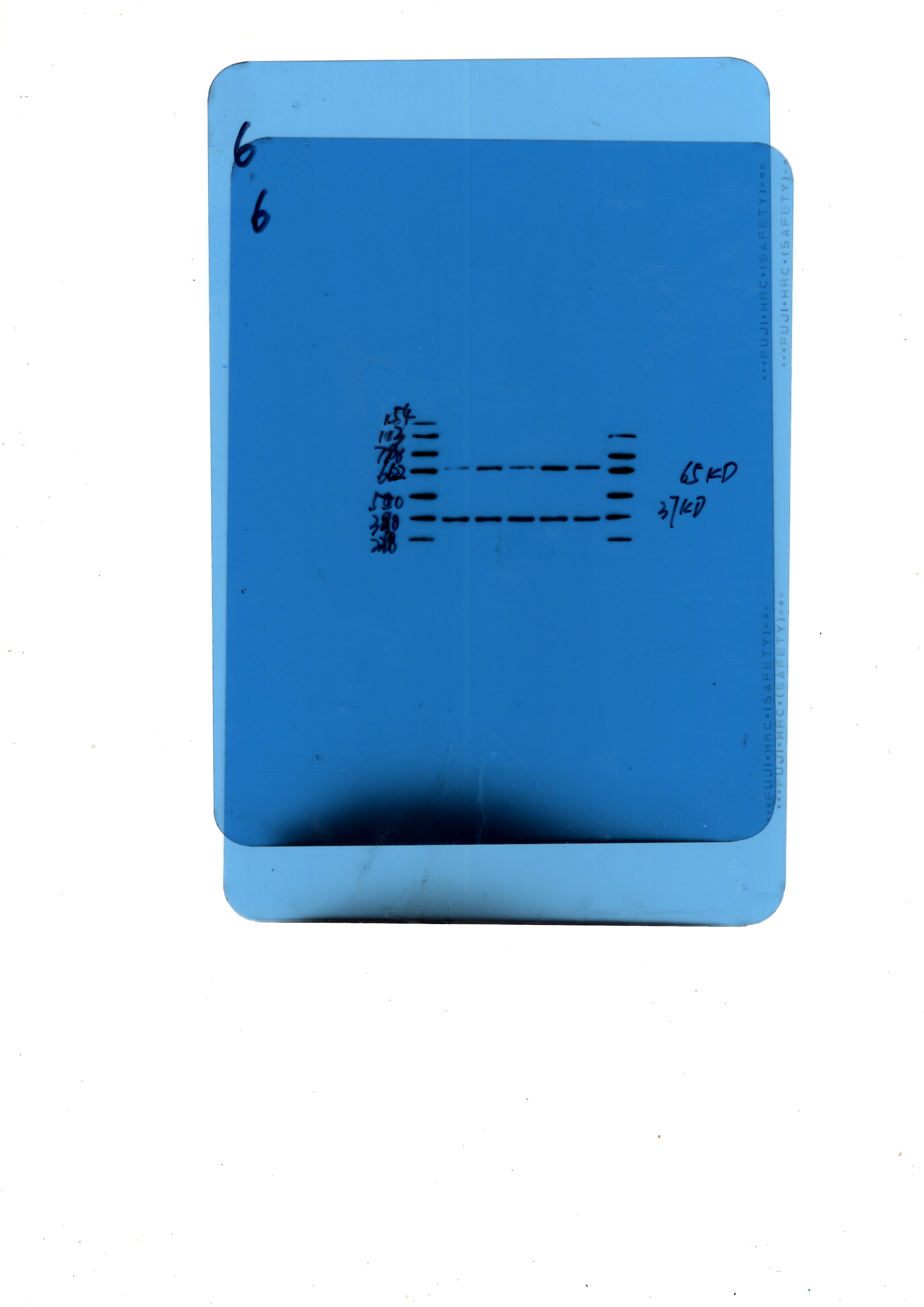

Supplement: Supplementary file 1 — Additional file 1. 3d 14d: Immunohistochemical results of PMMA group and ES-PMMA group at 3 and 14 days after surgery. 3d: Immunofluorescence results of PMMA group and ES-PMMA group at 3 days after surgery. 14d: Immunofluorescence results of PMMA group and ES-PMMA group at 14 days after surgery. NF-κB p65 1: The original blots/gels of NF-κB p65 (1). NF-κB p65 2: The original blots/gels of NF-κB p65 (2). NF-κB p65 3: The original blots/gels of NF-κB p65 (3). NF-κB p65 1: The original blots/gels of NF-κB p65 (1). p-NF-κB p65 -1: The original blots/gels of p-NF-κB p65 (1). p-NF-κB p65 -2: The original blots/gels of p-NF-κB p65 (2). p-NF-κB p65 -3: The original blots/gels of p-NF-κB p65 (3). TLR4 1: The original blots/gels of p-TLR4 (1). TLR4 2: The original blots/gels of p-TLR4 (2). TLR4 3: The original blots/gels of p-TLR4 (3). [file 13018_2023_3865_MOESM1_ESM.zip › Supplementary material/p-NF-a╩B p65 -3.jpg]

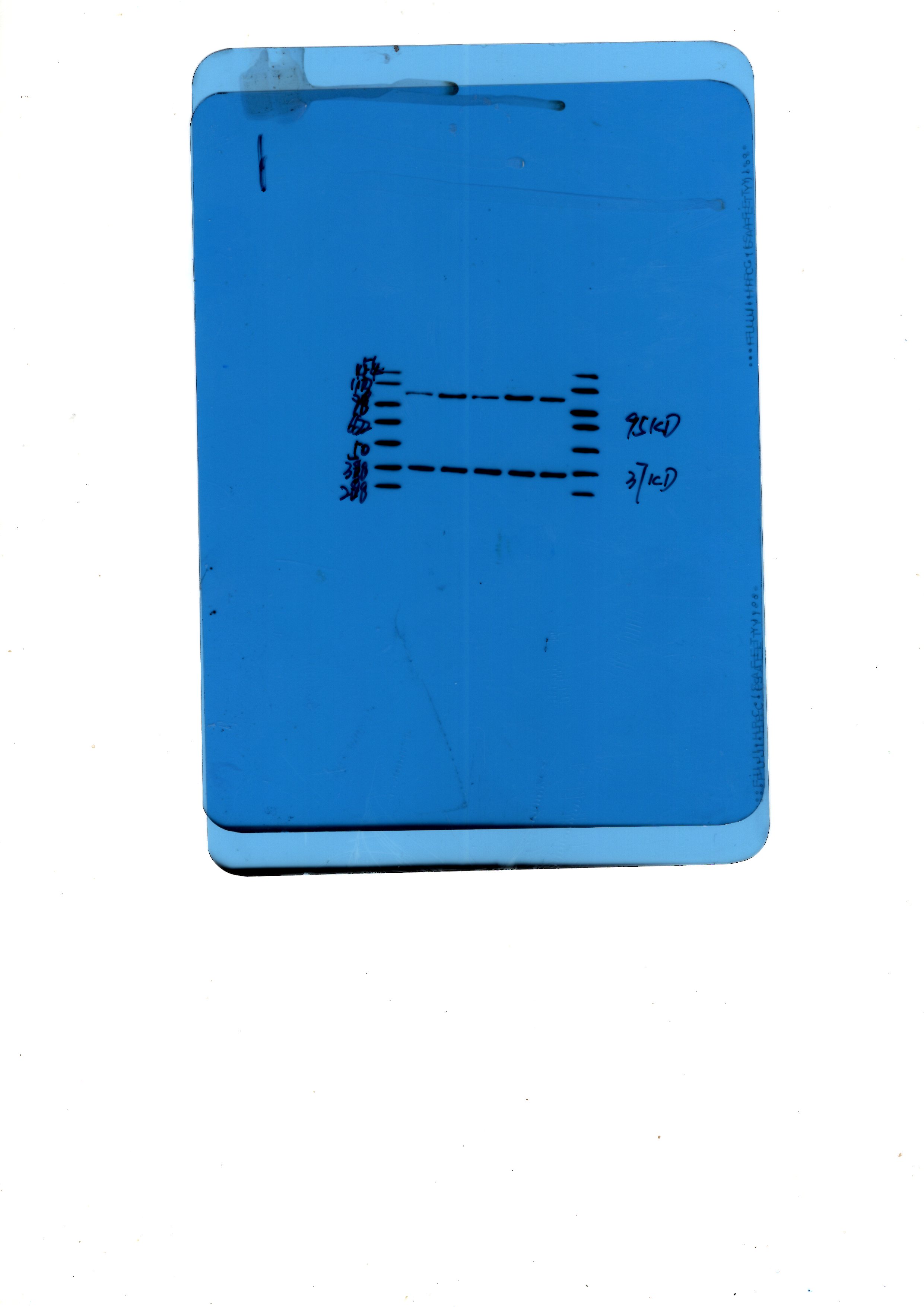

Supplement: Supplementary file 1 — Additional file 1. 3d 14d: Immunohistochemical results of PMMA group and ES-PMMA group at 3 and 14 days after surgery. 3d: Immunofluorescence results of PMMA group and ES-PMMA group at 3 days after surgery. 14d: Immunofluorescence results of PMMA group and ES-PMMA group at 14 days after surgery. NF-κB p65 1: The original blots/gels of NF-κB p65 (1). NF-κB p65 2: The original blots/gels of NF-κB p65 (2). NF-κB p65 3: The original blots/gels of NF-κB p65 (3). NF-κB p65 1: The original blots/gels of NF-κB p65 (1). p-NF-κB p65 -1: The original blots/gels of p-NF-κB p65 (1). p-NF-κB p65 -2: The original blots/gels of p-NF-κB p65 (2). p-NF-κB p65 -3: The original blots/gels of p-NF-κB p65 (3). TLR4 1: The original blots/gels of p-TLR4 (1). TLR4 2: The original blots/gels of p-TLR4 (2). TLR4 3: The original blots/gels of p-TLR4 (3). [file 13018_2023_3865_MOESM1_ESM.zip › Supplementary material/TLR4 1.jpg]

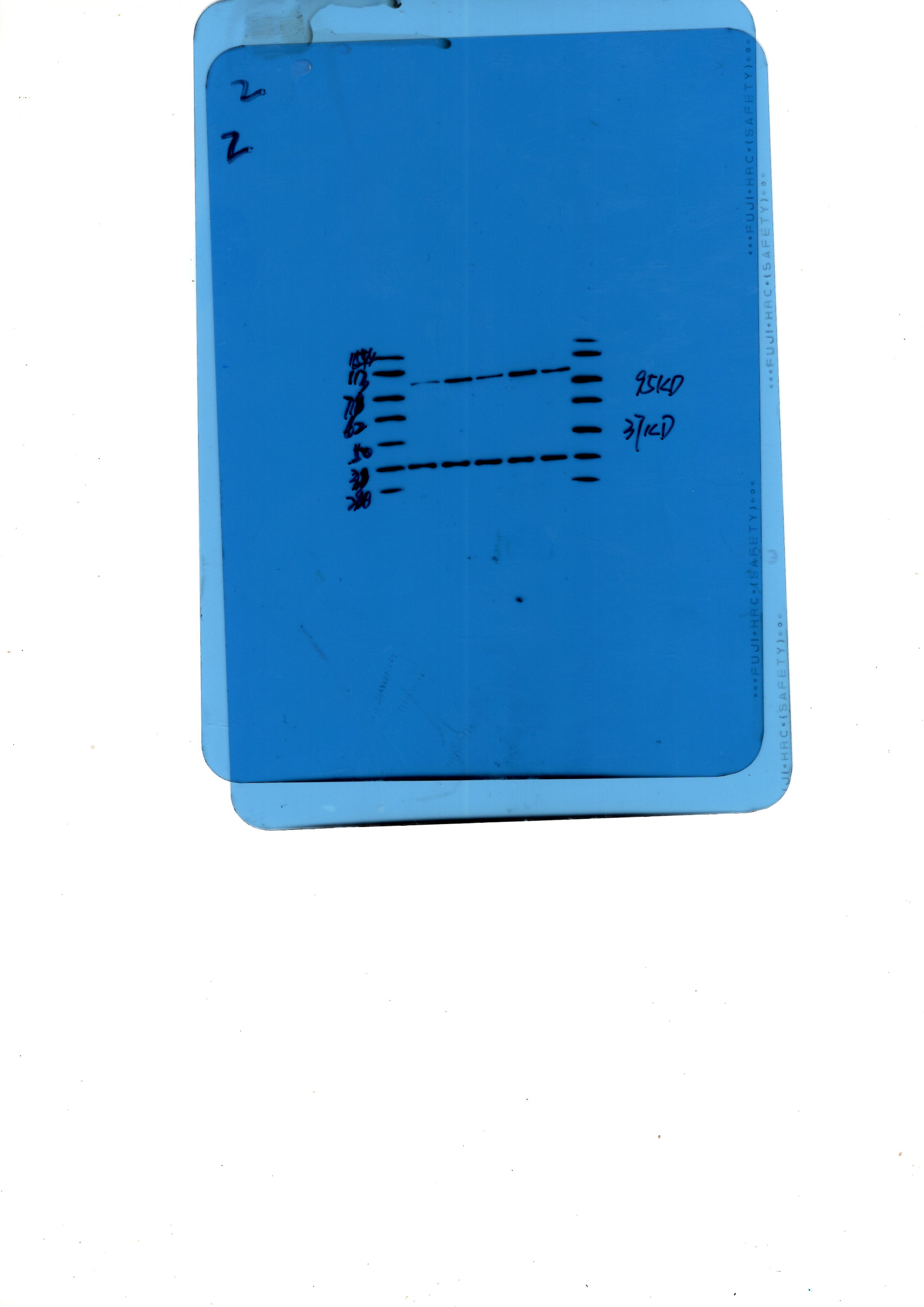

Supplement: Supplementary file 1 — Additional file 1. 3d 14d: Immunohistochemical results of PMMA group and ES-PMMA group at 3 and 14 days after surgery. 3d: Immunofluorescence results of PMMA group and ES-PMMA group at 3 days after surgery. 14d: Immunofluorescence results of PMMA group and ES-PMMA group at 14 days after surgery. NF-κB p65 1: The original blots/gels of NF-κB p65 (1). NF-κB p65 2: The original blots/gels of NF-κB p65 (2). NF-κB p65 3: The original blots/gels of NF-κB p65 (3). NF-κB p65 1: The original blots/gels of NF-κB p65 (1). p-NF-κB p65 -1: The original blots/gels of p-NF-κB p65 (1). p-NF-κB p65 -2: The original blots/gels of p-NF-κB p65 (2). p-NF-κB p65 -3: The original blots/gels of p-NF-κB p65 (3). TLR4 1: The original blots/gels of p-TLR4 (1). TLR4 2: The original blots/gels of p-TLR4 (2). TLR4 3: The original blots/gels of p-TLR4 (3). [file 13018_2023_3865_MOESM1_ESM.zip › Supplementary material/TLR4 2.jpg]

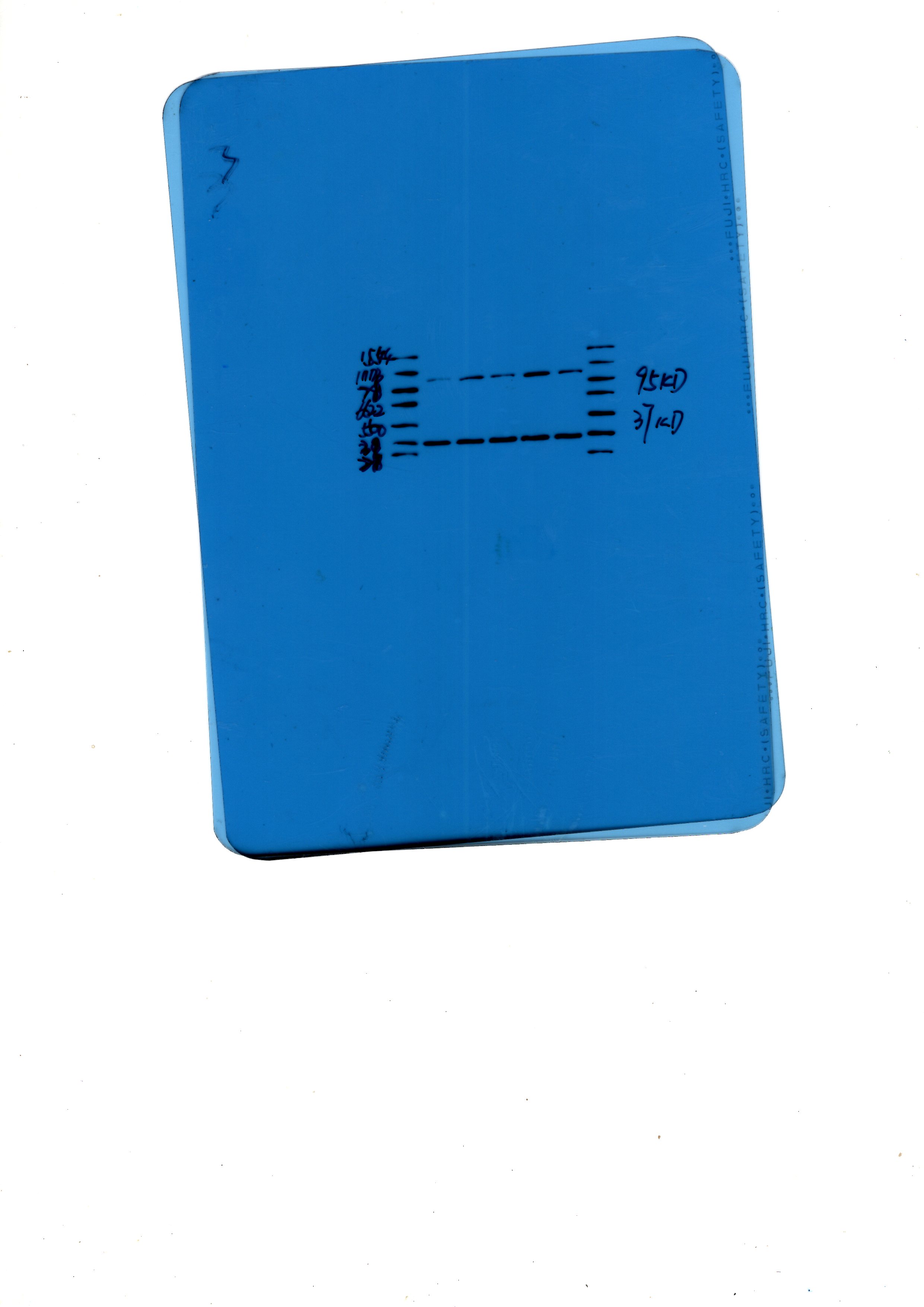

Supplement: Supplementary file 1 — Additional file 1. 3d 14d: Immunohistochemical results of PMMA group and ES-PMMA group at 3 and 14 days after surgery. 3d: Immunofluorescence results of PMMA group and ES-PMMA group at 3 days after surgery. 14d: Immunofluorescence results of PMMA group and ES-PMMA group at 14 days after surgery. NF-κB p65 1: The original blots/gels of NF-κB p65 (1). NF-κB p65 2: The original blots/gels of NF-κB p65 (2). NF-κB p65 3: The original blots/gels of NF-κB p65 (3). NF-κB p65 1: The original blots/gels of NF-κB p65 (1). p-NF-κB p65 -1: The original blots/gels of p-NF-κB p65 (1). p-NF-κB p65 -2: The original blots/gels of p-NF-κB p65 (2). p-NF-κB p65 -3: The original blots/gels of p-NF-κB p65 (3). TLR4 1: The original blots/gels of p-TLR4 (1). TLR4 2: The original blots/gels of p-TLR4 (2). TLR4 3: The original blots/gels of p-TLR4 (3). [file 13018_2023_3865_MOESM1_ESM.zip › Supplementary material/TLR4 3.jpg]
